# Systemic Bone Density at Disease Onset Is Associated With Joint Erosion Progression in Early Naive to Treatment Rheumatoid Arthritis: A Prospective 12-Month Follow-Up Open-Label Study

**DOI:** 10.3389/fmed.2021.613889

**Published:** 2021-02-25

**Authors:** Dario Bruno, Anna Laura Fedele, Barbara Tolusso, Angelina Barini, Luca Petricca, Clara Di Mario, Antonella Barini, Luisa Mirone, Gianfranco Ferraccioli, Stefano Alivernini, Elisa Gremese

**Affiliations:** ^1^Division of Rheumatology, Università Cattolica del Sacro Cuore, Rome, Italy; ^2^Division of Rheumatology, Fondazione Policlinico Universitario A. Gemelli IRCCS, Rome, Italy; ^3^Istituto di Biochimica e Biochimica Clinica, Fondazione Policlinico Universitario A. Gemelli IRCCS, Rome, Italy

**Keywords:** rheumatoid arthritis, osteoporosis, osteopenia, disease activity, biomarkers

## Abstract

**Objectives:** Osteoporosis and bone erosions are hallmarks of rheumatoid arthritis (RA) since disease onset is underpinned by the inflammatory burden. In this observational study, we aimed to dissect the putative RA-related parameters and bone-derived biomarkers associated with systemic and focal bone loss at disease onset and with their progression.

**Methods:** One-hundred twenty-eight patients with early rheumatoid arthritis (ERA) were recruited at disease onset. At study entry, demographic, clinical, and immunological parameters were recorded. Each ERA patient underwent plain X-rays of the hands and feet at study entry and after 12 months to assess the presence of erosions. After enrollment, each patient was treated according to the recommendations for RA management and followed up based on a treat-to-target (T2T) strategy. At baseline, blood samples for soluble biomarkers were collected from each patient, and plasma levels of osteoprotegerin (OPG), receptor activator of nuclear factor κB ligand (RANKL), Dickkopf-1 (DKK1), and interleukin 6 (IL-6) were assessed by enzyme-linked immunosorbent assay (ELISA). Seventy-one ERA patients underwent bone mineral density (BMD) measurement at the left femoral neck and second to fourth lumbar spine vertebrae (L2–L4) by dual-energy X-ray absorptiometry (DXA).

**Results:** Among the whole cohort, 34 (26.6%) ERA patients with bone erosions at study entry had a higher disease activity (*p* = 0.02) and IL-6 plasma levels (*p* = 0.03) than non-erosive ones. Moreover, at DXA, 33 (46.5%) ERA patients had osteopenia, and 16 (22.5%) had osteoporosis; patients with baseline bone erosions were more likely osteopenic/osteoporotic than non-erosive ones (*p* = 0.03), regardless of OPG, RANKL, and DKK1 plasma levels. Obese ERA patients were less likely osteopenic/osteoporotic than normal weight ones (*p* = 0.002), whereas anti-citrullinated protein antibodies (ACPA) positive ERA patients were more likely osteopenic/osteoporotic than ACPA negative ones (*p* = 0.034). At logistic regression analysis, baseline Disease Activity Score measured on 44 joints (DAS44) [OR: 2.46 (1.11–5.44)] and osteopenic/osteoporosis status [OR: 7.13 (1.27–39.94)] arose as independent factors of erosiveness. Baseline osteopenic/osteoporotic status and ACPA positivity were associated with bone damage progression during the follow-up.

**Conclusions:** Bone erosions presence is associated with systemic bone loss since the earliest phases of RA, suggesting that the inflammatory burden and autoimmune biology, underpinning RA, represent crucial enhancers of bone remodeling either locally as at systemic level.

## Introduction

Patients with rheumatoid arthritis (RA) present more bone loss than age- and sex-matched healthy controls (HC), regardless of treatment regimens (1). Traditionally, osteoporosis has been described as one of the common comorbidities linked to RA, with a prevalence of 10–50% depending on the studied population (2). Although bone loss may manifest in RA as juxta-articular loss and systemic bone loss (3), the most important feature is the focal bone loss as periarticular erosions, which are characterized by distinct differences in the pathogenesis. Given that in RA, a tight link exists between the inflammatory milieu and the bone remodeling process, a higher rate of bone loss should result as directly related to disease duration and disease activity. In early RA, where disease duration is limited, studying bone loss and bone erosions should give insights into the different mechanisms leading to the bone damage.

Therefore, RA is an excellent model to gain insights into the pivotal role of the immune system within the bone remodeling process involving multiple mechanisms. While bone surface erosions are due to the aberrant action of activated osteoclasts at sites of synovitis (4), the mechanisms guiding the juxta-articular and systemic bone loss and the decreases in bone mineral density (BMD) are less intuitive.

Osteoprotegerin (OPG), receptor activator of nuclear factor κB (RANK), and its ligand receptor activator of nuclear factor κB ligand (RANKL) play a central role (1, 5), involving members of the tumor necrosis factor (TNF) superfamily and its receptor and regulating the fine balance of bone resorption (6).

RANKL leads to the differentiation and activation of osteoclast precursors, by binding to surface RANK and determining the activation of nuclear factor κB and the transcription of osteoclastogenetic genes, with subsequent induction of preosteoclast differentiation, increased osteoclast activity, and prolonged their lifetime. Moreover, OPG is the soluble decoy receptor of RANKL, reducing osteoclastogenesis and bone resorption (1, 4, 6). In addition, the Wnt/β-catenin system is pivotal for bone mass regulation (7). In this pathway, the binding of Wnt-protein ligand to the surface Frizzled receptor induces an intracellular signaling cascade involving low-density lipoprotein receptor-related protein 5/6 (LRP-5/6), disheveled, and glycogen synthase kinase 3 that finally drives to β-catenin translocation into the nucleus leading to the upregulation of osteoblastic differentiation and survival gene transcription. Moreover, Dickkopf-1 (DKK1) acts as an endogenous antagonist of the Wnt pathway by interfering with LRP-5/6 phosphorylation (7).

Several studies have shown that osteoclasts activity and RANKL expression are risen at sites of RA synovitis (4, 8) and OPG acts as a bone loss protective factor in RANKL knockout mice despite active joint inflammation (6). Concerning DKK1, its plasma and synovial tissue levels are significantly increased in RA patients compared with HC, correlating with disease activity, supporting the putative critical role of DKK1 in bone erosions development in RA (7, 9).

Among the multiple factors promoting bone remodeling in RA, autoantibodies, as anti-citrullinated protein antibodies (ACPA) as well as anti-carbamylated peptide (anti-CarP) antibodies, have been associated with the development and progression of bone erosions and juxta-articular bone resorption as well as a decreased BMD (10–15). Therefore, systemic activators of bone remodeling and local osteoclastogenesis, in combination, lead to bone damage.

Besides autoantibodies, there have been advances made in understanding how inflammatory RA cytokines, such as TNF, IL-6, and interleukin 1 (IL-1), promote osteoclasts activation and inhibit osteoblasts function, interfacing with the complex system of bone turnover (1). Specifically, IL-6 acts as a bone resorbing cytokine by stimulating both osteoclasts precursor maturation and osteoblasts lineage expression of RANKL, in order to enhance osteoclastogenesis (16). Moreover, data from the ESPOIR cohort indicated that baseline IL-6 plasma levels were associated with structural damage over 3 years of follow-up, independently from disease activity and serological status (17).

Based on these issues, the aims of the present study were (i) to dissect the putative disease-related parameters (clinical and autoimmune) and bone-derived biomarkers associated with systemic and focal bone loss in terms of BMD and erosions at disease onset and (ii) to test their possible association with the progression of RA-related bone damage.

## Materials and Methods

### Patient Enrollment

In this single-center study, 128 patients, fulfilling the 2010 European League Against Rheumatism/American College of Rheumatology (EULAR/ACR) classification criteria for RA (18), were consecutively recruited between 2011 and 2019. Each RA patient had symptoms duration <12 months at study entry, and patients with disease duration <3 months were defined as “very early RA” (VERA) (19). At study entry, demographic, clinical, and immunological parameters were recorded, and each enrolled RA patient was naive to conventional disease-modifying antirheumatic drugs (cDMARDs) or biological DMARDs (bDMARDs). At enrollment, low dose steroid treatment (prednisone equivalent ≤5 mg/day) was allowed. After enrollment, each RA patient was treated according to the current recommendations for RA management (20), and at each study visit, the ACR/EULAR core data set [erythrocyte sedimentation rate (ESR), C-reactive protein (CRP), swollen joint count (SJC), tender joint count (TJC), physician and patient global assessment, pain, and Health Assessment Questionnaire (HAQ)] was recorded. Moreover, each enrolled RA patient was followed up based on a treat-to-target (T2T) strategy, with clinical assessment every 3 months recording the clinical improvement and remission based on the Disease Activity Score measured on 44 joints (DAS44) values (21, 22) and ACR/EULAR criteria, respectively (23). Twenty age- and sex-matched healthy subjects were included as the comparison group. The study protocol was approved by the Ethics Committee of the Università Cattolica del Sacro Cuore. All subjects provided signed informed consent.

### Detection of Autoantibodies

At study entry, immunoglobulin A (IgA)- and immunoglobulin M (IgM)-rheumatoid factor (RF) (Orgentec Diagnostika, Bounty, UK) and ACPA (Menarini, Italy) were assessed using the enzyme-linked immunosorbent assay (ELISA) or chemiluminescent methods, respectively. The cut-off levels were 20 U/mL for IgM-RF and IgA-RF and 5 U/mL for ACPA. Seronegative RA patients were defined as those negative for ACPA, IgM-RF, and IgA-RF; all the other subjects were described as seropositive.

### Assessment of Inflammatory and Bone Remodeling-Related Serum Biomarkers

At study entry, each enrolled RA patient underwent peripheral blood drawing after overnight fasting, and plasma was stored at −80°C until analysis. Bone turnover markers as OPG (sensitivity: 0.14 pmol/L; Biomedica, Austria), RANKL (sensitivity: 0.02 pmol/L; Biomedica, Austria), and DKK1 (sensitivity: 0.38 pmol/L; Biomedica, Austria) plasma levels and interleukin 6 (IL-6, sensitivity: 0.7 pg/ml, Bio-Techne, UK) plasma levels were detected by ELISA.

### Bone Damage Assessment

Each enrolled patient underwent plain X-rays of the hands and feet at study entry and after 12 months of follow-up. The presence of erosions was assessed, with an anterior and posterior view, using the van der Heijde modified Total Sharp Score (mTSS) (24) and the Larsen scores, respectively (25), by one experienced rheumatologist unaware of the patients' clinical and laboratory status. Erosive patients were defined by the presence of erosions. Moreover, the appearance of one new erosion (as a worsening of erosion score) was recorded at 12 months of follow-up, respectively.

### BMD Measurements

At study entry, seventy-one ERA patients underwent BMD measurement at the left femoral neck and second to fourth lumbar spine vertebrae (L2–L4) by dual-energy X-ray absorptiometry (DXA), using Lunar Prodigy equipment. A daily quality check of the DXA machine was done. A densitometric diagnosis of osteoporosis was based on BMD measurement according to the World Health Organization (WHO), and detected BMD was compared with the mean BMD of sex-matched young normal adults. In particular, considering the standard deviation (SD) from the mean peak bone mass (T score), osteoporosis status was defined as <-2.5 SD in at least one site, and osteopenia (low BMD) as a T score between −1.0 and −2.5 SD, whereas normal BMD was defined as a T score between +2.5 and −1 SD, respectively (26).

### Statistical Analysis

Statistical analysis was performed using SPSS version 21.0 (SPSS, Chicago, IL, USA) and Prism software (GraphPad, San Diego, CA, USA). Categorical and quantitative variables were described as frequency, percentage, and mean ± SD. Data on demographic and clinical features were compared between patients by the non-parametric Mann–Whitney U test or X^2^ test, as appropriate. Spearman's rank correlation test was used for correlation in all analyses. We performed a logistic regression model to determine the influence of the dependent variable “presence of bone erosions at disease onset” by the independent variables that reached the value of *p* < 0.10 at the univariate analysis. The values are expressed as odds ratio (OR) and 95% confidential interval (95% CI), respectively. A *p* < 0.05 was considered statistically significant.

## Results

### Baseline Bone Damage in ERA Patients Is Related to Disease Burden and Systemic Bone Loss Regardless of Bone-Derived Biomarkers

Demographic, clinical, and radiological characteristics of the enrolled ERA cohort at study entry are summarized in Table 1. Among the enrolled ERA cohort, 115 (89.8%) patients were women who did not differ based on age (53.03 ± 14.54 years) compared with men ERA patients (57.38 ± 16.34 years, *p* = 0.24).

**Table 1:** Demographic and clinical characteristics of ERA patients at diagnosis, according to erosiveness.

**Variable**	**ERA patients** ** (*N* = 128)**	**Erosive** ** (*N* = 34)** ** (26.6%)**	**Non-erosive** ** (*N* = 94)** ** (73.4%)**	***P***
Age (years)	53.4 ± 14.7	55.38 ± 14.4	52.79 ± 14.9	0.30
Gender, female (%)	115 (89.8)	29 (85.3)	86 (91.5)	0.31
Symptom duration (months)	5.62 ± 3.50	5.62 ± 3.43	5.62 ± 3.55	0.84
VERA (%)	45 (35.2)	10 (29.4)	35 (37.2)	0.41
Smokers (%)	38 (29.9)	9 (26.5)	29 (30.9)	0.60
Obese (BMI ≥ 30 kg/m^2^) (%)	16 (12.5)	4 (11.8)	12 (12.8)	0.88
Seropositive (%)	92 (71.9)	23 (67.6)	69 (73.4)	0.52
ACPA positive (%)	82 (64.1)	21 (61.8)	61 (64.9)	0.75
IgM-RF positive (%)	67 (52.3)	19 (55.9)	48 (51.1)	0.63
IgA-RF positive (%)	41 (32.0)	14 (41.2)	27 (28.7)	0.18
ESR (mm/1st h)	37.64 ± 23.82	38.59 ± 27.89	37.30 ± 22.32	0.74
CRP (mg/L)	16.44 ± 25.93	22.16 ± 38.02	14.33 ± 19.57	0.57
IL-6 (pg/mL)	20.74 ± 40.53	24.53 ± 32.01	19.52 ± 42.99	**0.03**
SJC44	8.73 ± 6.45	10.94 ± 7.06	7.94 ± 6.05	**0.03**
TJC44	12.94 ± 8.07	13.44 ± 7.73	12.76 ± 8.22	0.52
DAS44	3.53 ± 0.99	3.82 ± 1.15	3.43 ± 0.92	**0.02**
CDAI	26.67 ± 12.51	28.93 ± 13.98	25.84 ± 11.91	0.19
SDAI	28.32 ± 13.61	31.15 ± 15.15	27.27 ± 12.92	0.14
HAQ	1.09 ± 0.71	1.23 ± 0.82	1.04 ± 0.66	0.24

*Data are shown as mean ± SD or n (%). VERA, very early rheumatoid arthritis; BMI, body mass index; ACPA, anti-citrullinated protein antibodies; IgM, immunoglobulin M; IgA, immunoglobulin A; RF, rheumatoid factor; ESR, erythrocyte sedimentation rate; CRP, C-reactive protein; IL-6, interleukin-6; SJC44, 44 swollen joint count; TJC44, 44 tender joint count; DAS44, Disease Activity Score measured on 44 joints; CDAI, Clinical Disease Activity Index; SDAI, Simplified Disease Activity Index; HAQ, Health Assessment Questionnaire. Bold values indicate p <0.05*.

**Figure 1:**
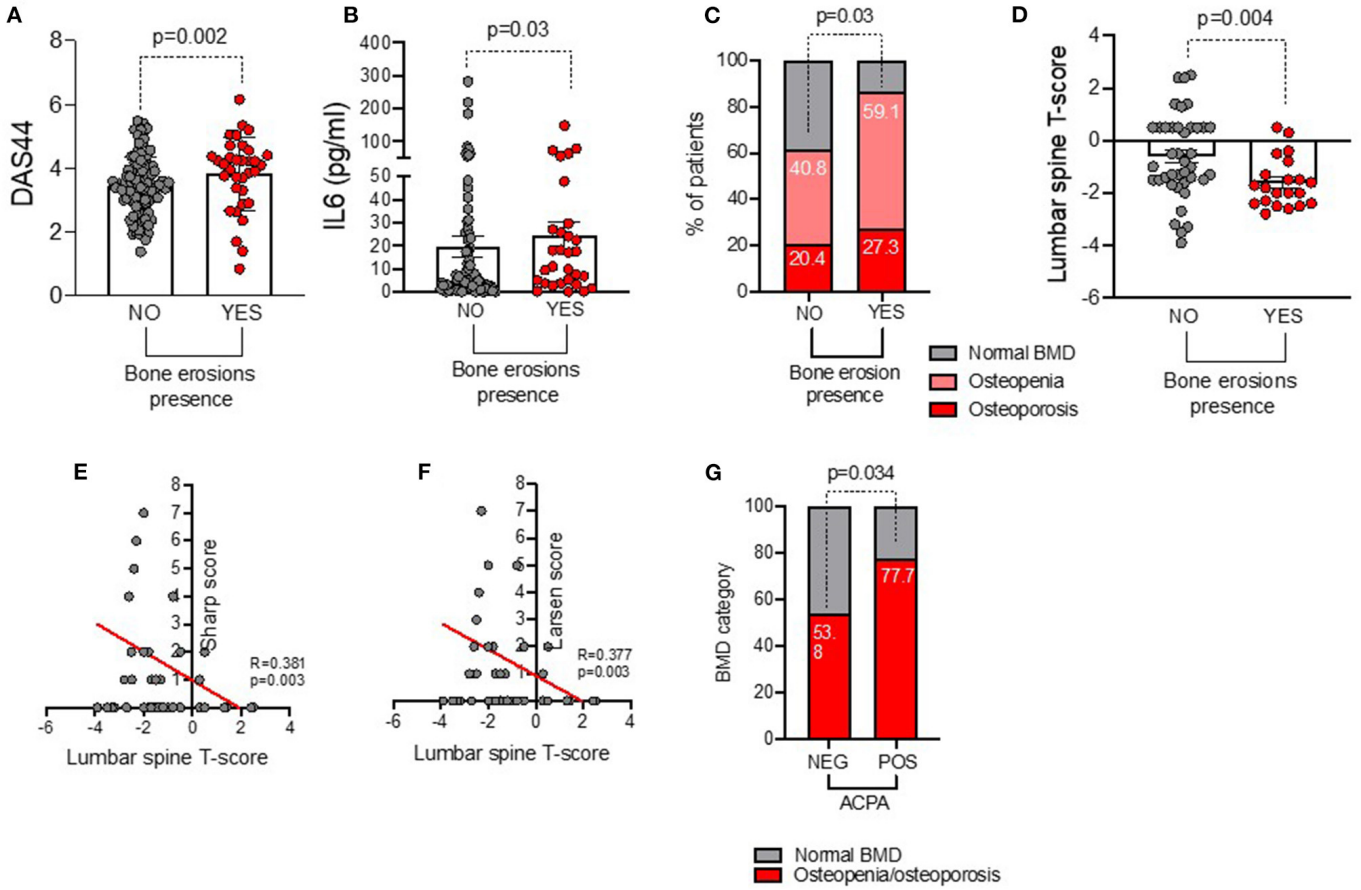
**(A–G)** Associations between baseline bone damage in ERA patients with disease burden and systemic bone loss. **(A)** DAS44 value in ERA patients stratified based on the presence of baseline bone erosions at plain radiographs of the hands and feet, Mann–Whitney U test. **(B)** IL-6 plasma levels in ERA patients stratified based on the presence of baseline bone erosions at plain radiographs of the hands and feet, Mann–Whitney U test. **(C)** Rate of systemic bone loss at baseline lumbar spine and femur DEXA in ERA patients (*n* = 71) stratified based on the presence of baseline bone erosions at plain radiographs of the hands and feet, X^2^ test. **(D)** Lumbar spine T score value in ERA patients stratified based on the presence of baseline bone erosions at plain radiographs of the hands and feet, Mann–Whitney U test. Correlation between lumbar spine T score value and Sharp **(E)** and Larsen **(F)** scores, Spearman rank correlation test. **(G)** Rate of osteopenia/osteoporosis in ERA patients at baseline stratified based on ACPA positivity, X^2^ test. A *p* < 0.05 was considered statistically significant. DAS44, Disease Activity Score measured on 44 joints; IL-6, interleukin-6; BMD, bone mineral density; DEXA, dual-energy X-ray absorptiometry.

Each ERA patient underwent plain radiographs of the hands and feet at study entry to evaluate bone erosions. Among the whole cohort, 34 (26.6%) ERA patients showed bone erosions. In particular, as shown in Figures 1A,B, erosive ERA patients had more likely a higher disease activity and inflammatory status as underlined by a significantly higher IL-6 plasma levels (24.53 ± 32.01 pg/ml) and DAS44 (3.82 ± 1.15) than non-erosive ERA patients (IL-6: 19.52 ± 42.99 pg/ml, *p* = 0.03 and DAS44: 3.43 ± 0.92, *p* = 0.02, respectively), regardless of ACPA positivity.

Among the whole ERA cohort, 71 patients underwent lumbar spine and femur DXA to investigate the rate of systemic bone loss (Supplementary Table 1). At study entry, 33 (46.5%) ERA patients had osteopenia, and 16 (22.5%) had osteoporosis, respectively. As shown in Figure 1C, ERA patients with baseline bone erosions were more likely osteopenic/osteoporotic at DXA (86.4%) than ERA patients without bone erosions (61.2%, *p* = 0.03).

In particular, at baseline, erosive ERA patients had a lower lumbar spine T score (−1.61 ± 0.94) than non-erosive ERA patients (−0.61 ± 1.60, *p* = 0.004) (Figure 1D). Lumbar T score at study entry inversely correlated with Sharp (R = −0.38, *p* = 0.003) and Larsen (R = −0.37, *p* = 0.003) scores (Figures 1E,F). Nevertheless, no significant association was detected between erosiveness and BMD measurement at the femoral neck in the same cohort. Interestingly, at study entry, obese ERA patients were less likely osteopenic/osteoporotic (36.4%) than normal weight ERA (84.4%, *p* = 0.002). Conversely, ACPA^pos^ ERA patients were more likely osteopenic/osteoporotic (77.7%) than ACPA^neg^ ERA patients (53.8%, *p* = 0.03) before any pharmacologic treatment (Figure 1G), whereas no significant association was found between IgA/IgM-RF positivity and baseline BMD in our ERA cohort (data not shown).

Considering the symptoms duration, patients with VERA did not differ in terms of baseline disease activity, IL-6 plasma levels, BMD measurement, or erosiveness rate compared with no VERA ones (data not shown).

Finally, at logistic regression model, baseline DAS44 [OR: 2.46 (1.11–5.44)] and osteopenic/osteoporosis status [OR: 7.13 (1.27–39.94)] arose as independent factors of erosiveness, whereas baseline IL-6 plasma levels were not independently associated with bone erosions presence in ERA.

### Bone-Derived Biomarkers Are Related to Clinical and Immunological Features in ERA Patients at Disease Onset

Since bone remodeling is an active process involving multiple soluble factors as DKK1, OPG, and RANKL, we investigated their expression in the peripheral blood of our ERA cohort in relation to the clinical and immunological features of the disease (including IL-6 plasma levels) as well as erosiveness.

**Figure 2:**
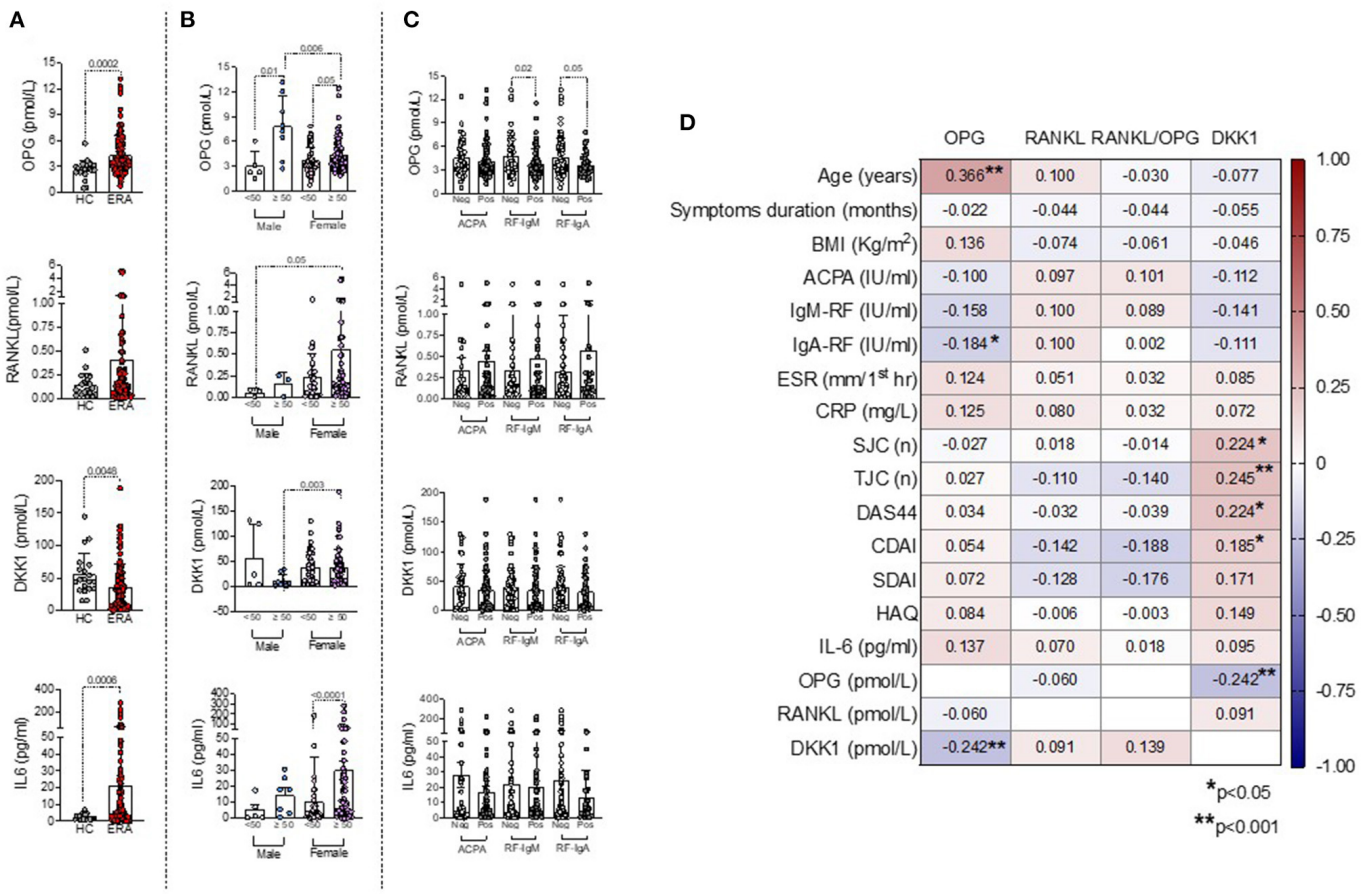
**(A–D)** Associations between bone-derived biomarkers with clinical and immunological features in ERA patients at disease onset. **(A)** OPG, RANKL, DKK1, and IL-6 plasma levels at baseline in ERA patients and HC, Mann–Whitney U test. **(B)** OPG, RANKL, DKK1, and IL-6 plasma levels at baseline in ERA patients stratified based on sex and age categories, Mann–Whitney U test. **(C)** OPG, RANKL, DKK1, and IL-6 plasma levels at baseline in ERA patients stratified based on individual ACPA, IgA/IgM-RF positivity, Mann–Whitney U test. **(D)** Heatmap showing the correlations between bone-derived biomarkers and RA features, Spearman rank correlation test. A *p* < 0.05 was considered statistically significant. ERA, early rheumatoid arthritis; OPG, osteoprotegerin; RANKL, receptor activator of nuclear factor kappa beta; DKK1, Dickkopf-1; ACPA, anti-citrullinated protein antibodies; IgA/IgM-RF, immunoglobulin A or immunoglobulin M isotypes rheumatoid factor; BMI, body mass index; ESR, erythrocyte sedimentation rate; CRP, C-reactive protein; SJC, swollen joint count; TJC, tender joint count; DAS44, Disease Activity Score measured on 44 joints; CDAI, Clinical Disease Activity Index; SDAI, Simple Disease Activity Index; HAQ, Health Assessment Questionnaire; IL-6, interleukin-6.

As shown in Figure 2A, ERA patients showed significantly higher plasma levels of IL-6 (21.04 ± 40.80 pg/ml) and OPG (4.31 ± 2.25 pmol/L) and lower plasma levels of DKK1 (37.02 ± 32.86 pmol/L) than HC (IL-6: 2.51 ± 1.63 pg/ml, *p* = 0.0006; OPG: 2.65 ± 2.11 pmol/L, *p* = 0.0002; DKK1: 55.48 ± 32.70 pmol/L, *p* = 0.0048), whereas RANKL plasma levels were comparable.

Moreover, women had significantly higher DKK1 plasma levels (37.99 ± 33.63 pmol/L) than men ERA (28.39 ± 45.04 pmol/L), whereas no differences were found for OPG and RANKL plasma levels (Supplementary Figure 1). However, stratifying ERA patients based on sex and age categories, women older than 50 years old showed significantly higher DKK1 (38.06 ± 35.79 pmol/L) plasma levels than men ERA older than 50 years old (10.86 ± 12.86 pmol/L, *p* = 0.003) and higher RANKL plasma levels (0.55 ± 1.14 pmol/L) than men ERA younger than 50 years old (0.06 ± 0.04 pmol/L, *p* = 0.05), respectively (Figure 2B). Conversely, regardless of sex, ERA patients older than 50 years old showed significantly higher OPG plasma levels (4.78 ± 2.05 pmol/L) than ERA patients younger than 50 years old (3.63 ± 1.79 pmol/L), mainly in men (Figure 2B). In line with these findings, among the bone-derived biomarkers, only OPG plasma levels directly correlated with age at diagnosis in ERA patients (R = 0.366, *p* < 0.0001) (Figure 2D). Moreover, stratifying ERA patients based on the autoimmune profile, autoantibody positivity *per se* at study entry was not related to OPG, RANKL, and DKK1 plasma levels (Figure 2C). However, considering the individual autoantibody positivities, OPG plasma levels were significantly increased in IgA-RF^neg^ (4.62 ± 2.49 pmol/L) and IgM-RF^neg^ (4.82 ± 2.51 pmol/L) ERA patients compared with seropositive ones (IgA-RF^pos^: 3.66 ± 1.44 pmol/L, *p* = 0.05 and IgM-RF^pos^: 3.85 ± 1.88; *p* = 0.02, respectively) (Figure 2C). ACPA positivity was not related to OPG, RANKL, or DKK1 plasma levels in ERA patients at study entry. Furthermore, DKK1 plasma levels directly correlated with disease activity parameters in ERA patients at disease diagnosis [DAS44: R = 0.224, *p* = 0.01 and Clinical Disease Activity Index (CDAI): R = 0.185, *p* = 0.04, respectively], whereas no correlations were found for OPG or RANKL plasma levels (Figure 2D). At enrollment, ERA patients under treatment with low-dose of steroid did not differ in terms of OPG, DKK1, or RANKL plasma levels compared with free of steroid treatment ERA patients (data not shown). Finally, in our cohort of ERA patients, we did not observe any significant difference in terms of OPG, RANKL, and DKK1 plasma levels based on the erosive status at disease onset (Supplementary Figure 2) and symptoms duration.

### Baseline Variables Associated With Bone Damage Progression in ERA Patients During Tight Control Scheme

**Figure 3:**
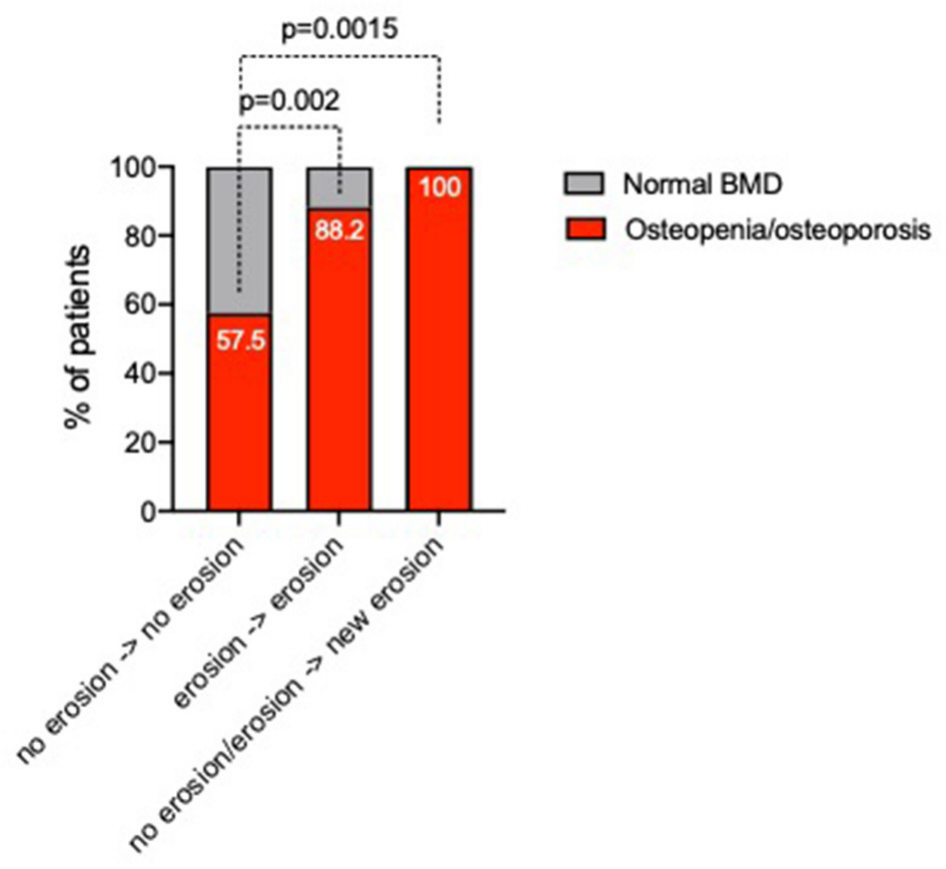
Rate of changes of bone damage across 12 months of follow-up in ERA patients stratified based on baseline BMD category. ERA patients were stratified as follows: no erosion at baseline and no bone damage progression, stable bone damage despite baseline erosion presence, and new development or worsening of bone erosions, respectively. A *p* < 0.05 was considered statistically significant. BMD, bone mineral density; ERA, early rheumatoid arthritis.

To assess the bone damage progression, 114 (89.1%) ERA patients underwent plain radiographs of the hands and feet at 12 months of follow-up within a T2T therapeutic approach. Particularly, 9 (7.9%) ERA patients experienced new onset or worsening of bone erosions. Of note in this time-frame, 25 (21.9%) ERA patients had begun a bDMARD. As shown in Figure 3, baseline BMD category was associated with bone damage progression in ERA patients since ERA patients experiencing new development or worsening of bone damage across the 12 months of follow-up were more likely osteopenic/osteoporotic (100%) than ERA patients not experiencing bone damage (57.5%, *p* = 0.0015). Moreover, neither disease activity and symptoms duration at baseline nor the rate of treatment response in terms of remission achievement was significantly different in ERA patients experiencing bone damage worsening compared with ERA patients without any sign of bone damage. Nevertheless, considering the autoimmune profile, 100% of ERA patients who experienced new bone erosions or their worsening within 12 months of follow-up were ACPA^pos^, whereas no significant differences of bone damage across 12 months of follow-up were found considering IgA- or IgM-RF positivity. Finally, considering the bone-derived biomarkers, ERA patients experiencing bone damage progression did not differ in terms of OPG, DKK1, and RANKL plasma levels compared with ERA patients not experiencing any bone damage progression (data not shown).

## Discussion

In this cohort of early RA, we provide evidence that systemic bone loss is a common finding in ERA, is linked to disease activity and ACPA positivity, and is an independent factor of bone damage at disease onset.

It is well-known that osteoporosis is one of the most common comorbidities in RA leading to an increased fracture risk (2, 3). Nevertheless, despite in long standing RA, osteoporosis may be considered as a natural consequence of disease burden, sustained glucocorticoid exposure, and decreased mobility due to functional joint impairment. In ERA patients, the pathogenetic process promoting focal and systemic bone remodeling and damage might be more complex. In this contest, it is reasonable to assume that bone damage occurring in ERA patients mainly results from inflammatory cytokines' detrimental action, able to stimulate both systemic and local osteoclasts activation (1). Among them, TNF and IL-6 play a crucial role in the regulation of osteoclasts differentiation inducing the expression of RANKL on osteoblasts membrane (27–30); in addition, IL-6 may act indirectly on bone homeostasis, enhancing and mediating bone resorption-inducing effects exerted by TNFα and IL-1 (1).

In our study, we enrolled consecutive ERA patients at disease onset without previous exposure to conventional or biological DMARDs in whom plasma levels of bone-derived biomarkers were assessed, finding that women older than 50 years old have higher DKK1 plasma levels than men older than 50 years old and higher RANKL plasma levels than men younger than 50 years old. Moreover, we found that in ERA patients, OPG plasma levels directly correlated with age at disease onset regardless of sex, as was previously demonstrated in healthy subjects (31) as well as in systemic diseases characterized by bone loss (32). This might reflect a compensatory self-defense mechanism against age-related osteoporosis. In addition, DKK1 plasma levels were found to mirror disease activity at ERA onset (i.e., DAS44 and CDAI), supporting the ESPOIR cohort previous data (7), despite no clear correlation emerged between DKK1 plasma levels at ERA onset and erosiveness or systemic bone loss.

Furthermore, considering the serological status, we found that OPG plasma levels were more likely increased in IgA/IgM-RF^neg^ than in seropositive ERA at baseline, thus supporting the well-known lower tendency to erosiveness of seronegative RA (33).

Concerning bone damage, 34 (26.6%) ERA patients had RA-related bone erosions at baseline plain radiographs, associated with a higher disease activity and inflammatory disease burden than non-erosive ERA patients. In particular, in our ERA cohort, erosive patients showed significantly higher IL-6 plasma levels and DAS44 than non-erosive patients, underlying the tight link between disease activity and bone loss (34).

Despite a direct correlation between bone-derived biomarkers, as DKK1, and bone erosions progression in ERA patients at disease onset (7), no data were available on the systemic bone loss equilibrium in such cohort. Therefore, in our study, ERA patients underwent lumbar spine and femur DXA to investigate the rate of BMD loss and its link with focal bone damage and clinical or immunological features of the disease. Interestingly, we observed that, at baseline, erosive ERA patients had a significantly lower lumbar spine T score than non-erosive ERA patients and baseline lumbar T score was inversely correlated with Sharp and Larsen radiological scores, whereas no significant associations were found with BMD measurement at the femoral neck. These findings are in line with the ones previously reported by Hafez and colleagues (35) and are probably related to the increased presence of the trabecular bone at lumbar spine level (compared with the hip), which might be more sensitive to the inflammatory cytokine RA milieu, due to its increased metabolism (35). Interestingly, we found that erosive ERA patients were more likely osteoporotic than non-erosive ERA patients, despite no significant differences in terms of OPG, RANKL, and DKK1 plasma levels.

Among putative environmental and immunological disease-related factors that may influence BMD measurement in RA patients, we considered the body mass index (BMI) that might underestimate osteopenia/osteoporosis prevalence (36). As previously observed in the normal population (36), stratifying ERA patients based on their BMI, obesity was related to less incidence of osteopenia/osteoporosis at disease onset as suggested by the significantly higher plasma levels of IL-1 receptor antagonist in obese naive to treatment RA patients, which might antagonize the potent osteoclastogenic effects of IL-1β (37). Moreover, at baseline, ACPA^pos^ ERA patients, naive to any pharmacological treatment, had more likely osteopenia/osteoporosis of the lumbar spine and femur than ACPA^neg^ ERA patients, as previously demonstrated (38).

Nevertheless, little is known about the possible prognostic biomarkers of bone damage progression to be used at the first medical assessment within ERA patients management (34, 38). In our study, we investigated disease-related biomarkers possibly enabling the identification of ERA patients at higher risk of bone damage progression. In particular, after adopting a T2T approach, nearly 8% of patients had progression of focal bone damage, in terms of new erosions development or their worsening. Among baseline variables associated with bone damage progression, ERA patients experiencing new development or worsening of bone damage across 12 months of follow-up were more likely ACPA^pos^ than ERA patients without bone damage worsening or development (38). Therefore, we fully confirm that ACPA positivity is the most important prognostic risk factor for baseline systemic bone loss and future erosions despite a T2T strategy in ERA patients (38).

## Conclusion

In summary, our study confirms that focal bone damage (bone erosions) is associated with systemic bone loss (osteopenia/osteoporosis) since the earliest phases of RA, suggesting that the inflammatory burden and the autoimmune biology, underpinning RA, represent crucial enhancers of bone remodeling either at the local as at the systemic level. Thus, early RA is a pathognomonic model to define the molecular mechanisms of systemic and local bone damage progression.

## Data Availability Statement

The raw data supporting the conclusions of this article will be made available by the authors, without undue reservation.

## Ethics Statement

The studies involving human participants were reviewed and approved by Ethical Committee of the Università Cattolica del Sacro Cuore. The patients/participants provided their written informed consent to participate in this study.

## Author Contributions

DB, AF, and EG: conceived the study. DB, AF, BT, AngB, LP, CD, AntB, LM, GF, and SA: collected the clinical data. DB, AF, BT, AngB, CD, and AntB: performed the experiments. DB, BT, SA, and EG: performed the statistical analysis. DB, AF, BT, AngB, LP, CD, AntB, LM, GF, SA, and EG: drafted and revised the manuscript. All authors contributed to the article and approved the submitted version.

## Conflict of Interest

The authors declare that the research was conducted in the absence of any commercial or financial relationships that could be construed as a potential conflict of interest.
