# Supplementary material for: Systemic Bone Density at Disease Onset Is Associated With Joint Erosion Progression in Early Naive to Treatment Rheumatoid Arthritis: A Prospective 12-Month Follow-Up Open-Label Study
Source: Front Med (Lausanne). 2021 Feb 25;8:613889. doi: 10.3389/fmed.2021.613889 (PMC7959810; doi:10.3389/fmed.2021.613889)
Supplement: Supplementary file 1 [file Data_Sheet_1.pdf]

Supplementary Material

Supplementary Table 1. Demographic and clinical characteristics of ERA patients at diagnosis, according to BMD at DEXA.

	Bone Mineral Density (BMD)		
**Variable**	**Normal BMD (N = 22) (31.0 %)**	**Osteopenic or osteoporotic (N = 49) (69.0 %)**	**P**
**Age (years)**	53.91 ± 2.36	61.20 ± 1.39	**0.007**
**Gender, female (%)**	19 (86.4)	47 (95.9)	0.169
**Symptom duration (months)**	5.36 ± 0.71	5.78 ± 0.48	0.630
**VERA (%)**	9 (40.9)	16 (32.7)	0.341
**BMI (Kg/m^2)**	28.08 ± 0.79	24.34 ± 0.51	**0.001**
**Obese (BMI ≥ 30 kg/m2) (%)**	7 (31.8)	4 (8.2)	**0.011**
**Erosive, n(%)**	3 (13.6)	19 (38.8)	**0.029**
**Seropositive (%)**	13 (59.1)	36 (73.5)	0.175
**ACPA positive (%)**	10 (28.6)	35 (71.4)	**0.034**
**IgM-RF positive (%)**	11 (50.0)	26 (53.1)	0.507
**IgA-RF positive (%)**	5 (22.7)	16 (32.7)	0.290
**ESR (mm/1st h)**	45.14 ± 5.43	37.96 ± 3.27	0.211
**CRP (mg/L)**	0.77 ± 0.91	0.67 ± 0.10	0.644
**IL-6 (pg/mL)**	21.99 ± 9.38	34.00 ± 8.04	0.195
**SJC44**	9.45 ± 1.41	9.61 ± 0.98	0.975
**TJC44**	12.91 ± 1.96	13.86 ± 1.07	0.459
**DAS**	3.72 ± 0.22	3.65 ± 0.12	0.645
**HAQ**	1.28 ± 1.63	1.13 ± 0.10	0.537
**Sharp score**	0.64 ± 0.50	1.57 ± 0.46	**0.041**
**Larsen Score**	0.55 ± 0.42	1.51 ± 0.44	**0.039**
**DAS44 remission at 3 months FU, n(%)**	6 (31.6)	9 (19.6)	0.232
**DAS44 remission at 6 months FU, n(%)**	8 (38.1)	20 (40.8)	0.524
**DAS44 remission at 12 months FU, n(%)**	8 (36.4)	17 (35.4)	0.572
**Joint damage progression at 12 months FU, n(%)**	2 (9.1)	21 (42.9)	**0.005**

Data are shown as mean ± s.e.m. or n (%). **VERA**, very early Rheumatoid Arthritis; **BMI**, body mass index; **ACPA**, Anti-Citrullinated Protein Antibodies; **IgA/IgM-RF:** Immunoglobulin-A or Immunoglobulin M isotypes Rheumatoid Factor; **ESR**, Erythrocyte Sedimentation Rate; **CRP**, C-Reactive Protein; **IL-6**, interleukin-6; **SJC44**, swollen joint count over 44 joints; **TJC44**, tender joint

count over 44 joints; **DAS44**, Disease Activity Score measured on 44 joints; **HAQ**, Health Assessment Questionnaire.

Supplementary Figure 1

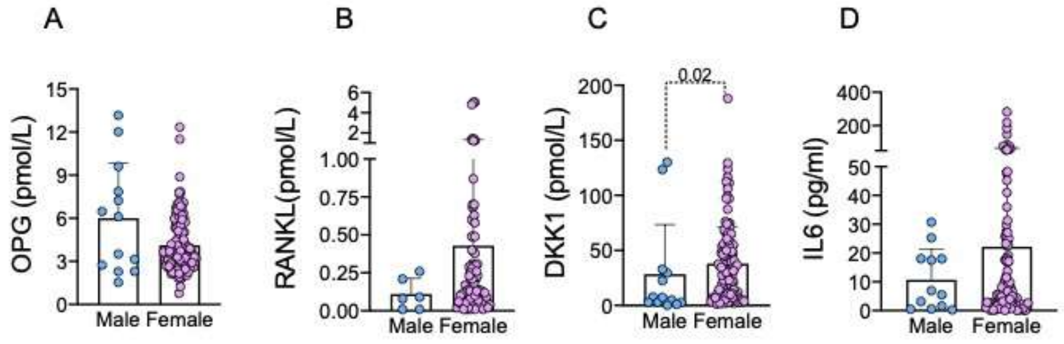

Supplementary Figure 1(A-D). Plasma levels of bone-derived biomarkers and IL-6 in ERA patients based on gender. (A) OPG **(B)** RANKL **(C)** DKK1 and **(D)** IL- 6plasma levels in ERA patients stratified based on gender. A $p < 0.05$ was considered statistically significant. **ERA:** Early Rheumatoid Arthritis; **OPG:** Osteoprotegerin; **RANKL:** receptor activator of nuclear factor kappa beta; **DKK1:** Dickkopf1.

Supplementary Figure 2

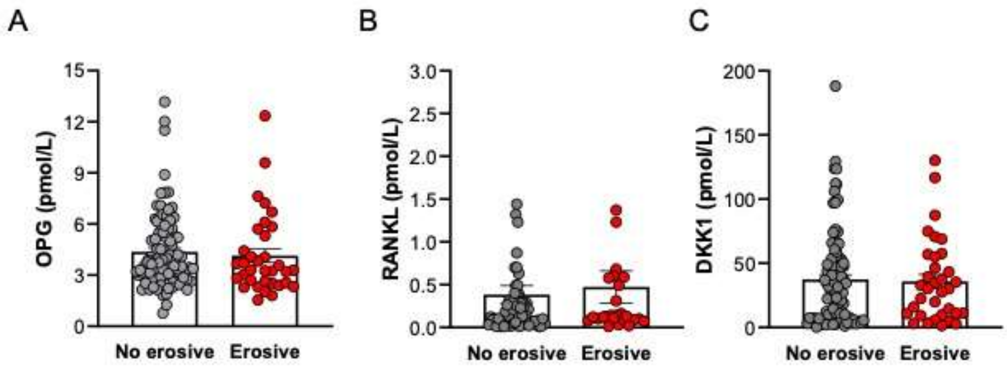

Supplementary Figure 2(A-C). Plasma levels of bone-derived biomarkers in ERA patients based on baseline presence of bone erosions. (A) OPG **(B)** RANKL and **(C)** DKK1 plasma levels in ERA patients stratified based on the presence of baseline bone erosions at plain radiographs of hands and feet. **ERA:** Early Rheumatoid Arthritis; **OPG:** Osteoprotegerin; **RANKL:** receptor activator of nuclear factor kappa beta; **DKK1:** Dickkopf1.
